# Evolving Patterns of Metastasis in Renal Cell Carcinoma: Do We Need to Perform Routine Bone Imaging?

**DOI:** 10.15586/jkcvhl.v8i4.202

**Published:** 2021-10-13

**Authors:** Justin Lin, Yue Zhang, Wei Hou, Qian Qin, Matthew D. Galsky, William K. Oh, Che-Kai Tsao

**Affiliations:** 1 Division of Hematology and Medical Oncology, The Tisch Cancer Institute, Icahn School of Medicine at Mount Sinai, New York, NY, USA;; 2 Division of Hematology and Medical Oncology, State University of New York at Stony Brook, New York, NY, USA;; 3 Department of Preventive Medicine, State University of New York at Stony Brook, New York, NY, USA

**Keywords:** bone imaging, immune checkpoint inhibitors, metastatic distribution, renal cell carcinoma, tyrosine kinase inhibitors

## Abstract

Advance diagnostic and treatment modalities have improved outcomes for renal cell carcinoma (RCC) patients, but the prognosis for those with metastatic disease (mRCC) remains poor. As given metastatic distribution is critical in guiding treatment decisions for mRCC patients, we evaluated evolving metastatic patterns to assess if our current practice standards effectively address patient needs. A systematic literature review was performed to identify all publicly available prospective clinical trials in metastatic renal cell carcinoma (mRCC) from 1990 to 2018. A total of 16,899 mRCC patients from 127 qualified phase I–III clinical trials with metastatic site documentations were included for analysis for incidence of metastases to lung, liver, bone, and lymph nodes (LNs) over time. Studies were categorized into three treatment eras based on the timing of regulatory approval: Cytokine Era (1990-2004), vascular endothelial growth factor/tyrosine kinase inhibitor (TKI) Era (2005-2016), and immune checkpoint inhibitor/TKI Era (ICI-TKI, 2017-2018) and also classified as first-line only (FLO) or second-line and beyond (SLB). Overall, an increase in the incidence of bone and LNs metastases in FLO and SLB, and lung metastases in FLO, was seen over the three treatment eras. Generally, the burden of disease is higher in SLB when compared with FLO. Importantly, in the ICI-TKI era, the incidences of bone metastasis are 28% in FLO and 29% in SLB settings. The disease burden in patients with mRCC has increased steadily over the past three decades. Given the unexpectedly high rate of bone metastasis, routine dedicated bone imaging should be considered in all patients with mRCC.

## Introduction

Kidney cancer is the eighth most common cancer in the United States, with an estimated 76,080 new cases in 2021 (1). Approximately 35% of renal cell carcinoma (RCC) eventually becomes metastatic (mRCC), with the most common sites being lung, liver, bone, and lymph nodes (LNs) (1–3). As diagnostic imaging and systemic treatments for mRCC improve the detection of metastatic sites and biology of the disease, also evolve. These can impact the selection, timing, and sequence of the present and future therapies.

Diagnostically, computed tomography (CT) has long been the modality of choice, with much improvement in its resolution and reproducibility over the last three decades (4). Increasing utilization of CT, magnetic resonance imaging (MRI), and positron emission tomography (PET)-CT scans have led to earlier detection and a higher incidence of RCC (5). The Response Evaluation Criteria in Solid Tumors (RECIST) criteria for solid tumors has also undergone revisions with impacts on the definition of mRCC and thus patient eligibilities for clinical trials (6). Compared with the RECIST 1.0 (2000), reporting of metastatic disease in RECIST 1.1 (2009) changed in several ways: the number of target lesions decreased from 5 to 2 per organ/10 to 5 total, LN assessment from no clear definition to specific measurements, PET scan was included for detection of new lesions, and a bone lesion >1 cm with soft tissue component was updated to count as a measurable disease. Additionally, routine bone imaging is not considered a standard practice in the absence of symptoms or associated laboratory abnormalities (7). However, the presence of osseous involvement may go undetected in routine body CT.

Several landscape changes have also occurred in the treatment of mRCC. Before 2005, cytokines such as interferon and interleukin-2 were considered standard treatment options. Vascular endothelial growth factor inhibitors (VEGFs) and tyrosine kinase inhibitors (TKIs) have since gained rapid Food and Drug Administration approval and replaced cytokines as the standard of care for advanced and mRCC (8–11). Most recently, immune checkpoint inhibitors (ICIs) with or without TKIs have ushered in the new era of therapeutic possibilities (12–18).

We hypothesize that the trend of metastatic site distribution in mRCC patients is evolving with improved diagnostics and therapeutic advancement. We aimed to characterize the pattern of metastases in mRCC patients through a systematic analysis of prospective clinical trials published. A better understanding of the evolving metastatic patterns will guide the practice standards that will effectively address the current patient needs.

## Materials and Methods

### Data extraction

A systematic literature review was performed to identify all publicly available, prospective clinical trials in mRCC from January 1990 to August 2018. Specifically, an independent review of citations from the PubMed database was conducted. The search included the keywords “renal cell carcinoma,” “kidney cancer,” and/or “metastatic renal cell carcinoma” and was limited to phase I–III clinical trials. In addition, Google Scholar citation was searched and manually reviewed to ensure no additional clinical trials could be identified. The computer search was supplemented with a manual review of the retrieved articles to establish accuracy. To confirm all eligible clinical trials were included, the website, www.ClinicalTrials.gov, was also searched manually. Duplicates were disregarded, and in the event of multiple publications within the same clinical trial, the most recent, complete, and updated version was included in this meta-analysis.

### Patient selection

All patients in the studies that fit the criteria of phase I–III clinical trial published between January 1990 to August 2018, only recruited mRCC patients and had clear documentation of metastatic distribution were included in the analyses. Only distant metastasis (M1) was included in this analysis, and Stage IV was defined based on the American Joint Commission on Cancer criteria. Studies were excluded if patients with other cancers were reported, did not document metastases in the baseline characteristics, were retrospective studies, reviews, meta-analysis, and/or non-English publications.

The patients fitting the inclusion criteria were analyzed and grouped into Cytokine Era (1990–2004), VEGF-TKI Era (2005–2016), and ICI-TKI Era (2017–2018) based on the standard of care treatments offered in the respective time frames. Patients’ baseline characteristics were classified into first-line only (FLO), second-line and beyond (SLB), or mixed (MIX, if the line of therapy was unclear at the time of metastatic sites documentation) settings.

### Statistical analysis

The primary outcomes of this study were percentages of metastases to lung, liver, bone, and LNs. The proportions of metastases were calculated using the numbers of metastases according to lines of systemic treatments – FLO and SLB, respectively, for each included study. Overall percentages were calculated using Equation (1).

$$\text{Overall percentages =}\frac{\begin{array}{l} \text{Sums of the numbers of metastases}\\ \text{across all studies} \end{array}}{\text{total numbers}}\;\left(1\right)$$

When a study had the MIX setting and did not report FLO and SLB separately, the numbers of metastases were estimated using Equation (2).

$$\begin{array}{l} \text{Number of metastases}=\text{Total number of}\\ \text{metastases of that study}\times \text{percentages of FLO}\\ \text{and SLB to the total sample size }\left(2\right) \end{array}$$

The percentages of metastasis were calculated for each era and compared using Chi-square tests. A “clean” analysis was performed with the exclusion of the patients in the MIX setting.

## Results

### Search results

**Figure 1:**
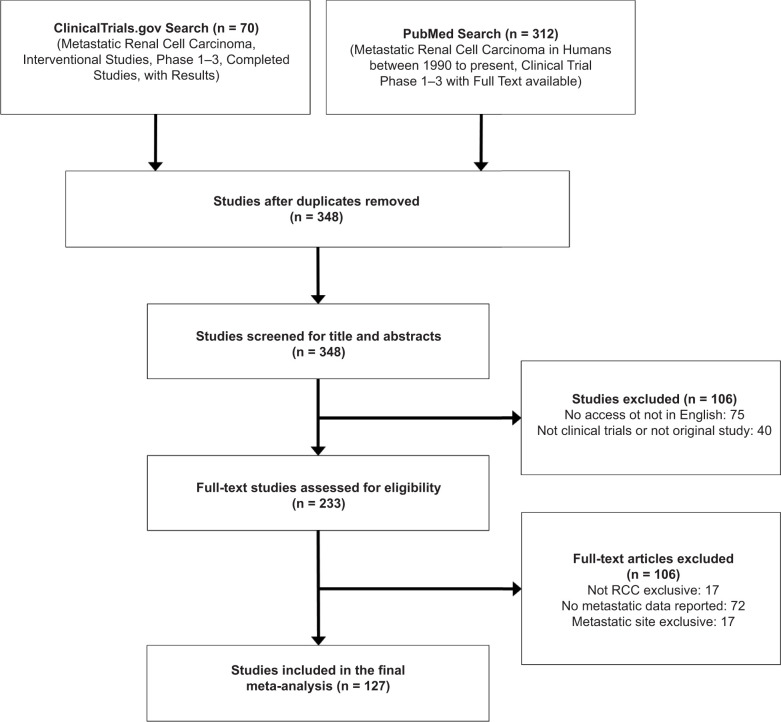
Study selection flow chart.

The literature search yielded 348 potential publications on mRCC. Excluding review articles, duplicate studies, non-English publications, editorials, meta-analyses, and observational studies, a total of 233 full-text studies were further reviewed. Excluding studies where metastatic site documentations were unclear/unavailable, 127 studies encompassing 16,899 mRCC patients were included in the final meta-analysis (Figure 1). Characteristics of these studies are listed in Table 1. Lung, liver, bone, and LN metastases were identified and grouped into the three predefined therapeutic timeframes.

**Table 1:** Included studies based on the treatment eras.

	Cytokine Era(1990–2004)N (%)	VEGF-TKI Era (2005–2016)N (%)	ICI- TKI Era (2017–2018)N (%)	TotalN (%)
FLO	11 (39%)	36 (41%)	7 (58%)	54 (43%)
SLB	4 (14%)	30 (34%)	4 (33%)	38 (30%)
Mixed	13 (46%)	21 (24%)	1 (8%)	35 (28%)
	Three reported metastatic numbers separately for FLO and SLB.	Three reported metastatic numbers separately for FLO and SLB.	None reported metastatic numbers separately for FLO and SLB.	
Total	28	87	12	127 (100%)

VEGF-TKI, vascular endothelial growth factor/tyrosine kinase inhibitor; ICI-TKI, immune checkpoint inhibitor/tyrosine kinase inhibitor; N, number of patients; FLO, first-line only; SLB: second line and beyond.

### Distribution of metastatic sites by a line of therapy within each treatment era

Detailed analyses of each era are subdivided into FLO versus SLB and outlined in Table 2. In the Cytokine Era, more bone metastasis was identified in the FLO compared with the SLB setting (23% vs. 15%; P = 0.0014). There were similar rates of LN (39% vs. 40%; P = 0.7773) and visceral involvements (lung: 58% vs. 65%; P = 0.0501 and liver: 18% vs. 22%; P =0.0863) between the two settings. In the VEGF-TKI Era, liver and LN involvements were significantly higher in the SLB setting than the FLO setting (liver: 25% vs. 20%; P < 0.0001 and LN: 50% vs. 46%; P = 0.0003). Bone and lung were stable with 25% and 70%, respectively. In the ICI-TKI Era, more LN involvement was identified in the SLB setting (44% vs. 57%; P = 0.0020). Rates of metastatic diseases in the lung (69% vs. 69%), liver (18% vs. 23%), and bone (28% vs. 29%) were similar.

**Table 2:** Percentage of metastasis by treatment era for all included studies (N=127).

Metastasis	Cytokine Era(1990–2004)			VEGF-TKI Era(2005–2016)			ICI-TKI Era(2017–2018)			P value^b^	P value^c^
	FLO	SLB	P value^a^	FLO	SLB	P value^a^	FLO	SLB	P value^a^		
Lung	58 %	65 %	0.0501	70 %	71 %	0.3037	69 %	69 %	0.9551	<.0001	0.2120
Liver	18 %	22 %	0.0863	20 %	25 %	<.0001	18 %	23 %	0.1480	0.0578	0.3340
Bone	23 %	15 %	0.0014	24 %	25 %	0.0777	28 %	29 %	0.7244	0.0002	<.0001
LN	39 %	40 %	0.7773	46 %	50 %	0.0003	44 %	57 %	0.0020	<0.0001	0.0051

VEGF-TKI, vascular endothelial growth factor/tyrosine kinase inhibitor; ICI-TKI, immune checkpoint inhibitor/tyrosine kinase inhibitor; N, number of patients; FLO, first-line only; SLB: second line and beyond; LN, lymph node.

a P value based on Chi-square tests comparing % metastasis between FLO and SLB within each era.

b P value based on Chi-square tests comparing % metastasis in FLO among the three eras.

c P value based on Chi-square tests comparing % metastasis in SLB among the three eras.

### Distribution of metastatic sites by a line of therapy over the treatment eras

In the FLO setting, there were a significant increase of disease in the lung (58% vs. 70% vs. 69%; P < 0.0001), bone (23% vs. 24% vs. 28%; P = 0.0002), and LNs (39% vs. 46% vs. 44%; P = 0.0002) with time. Liver disease detection was relative stable (22% vs. 25% vs. 23%; P = 0.0578) through the years. Higher detection of bone disease (15% vs. 25% vs. 29%; P < 0.0001) and LNs (40% vs. 50% vs. 57%; P = 0.0051) with time was observed in the SLB setting. The distributions of metastases were relative stable in the lung (65% vs. 71% vs. 69%; P = 0.2135) and liver (22% vs. 25% vs. 23%; P = 0.3340).

## Discussion

This study represents the largest analysis of metastatic sites for patients with mRCC to date, examining the patterns of metastasis in 16,899 patients derived from 127 prospective, mRCC clinical trials between 1990 and 2018. Specifically, the overall burden of metastatic disease has increased over time, especially when evaluated concerning the three distinct treatment eras: Cytokine, VEGF-TKI, and ICI-TKI is consistent with the initial hypothesis. First, changes in clinical trial reporting and advances in diagnostic imaging have contributed to the better definition and capturing of metastatic disease in this study population. Furthermore, as survival improves with better therapies, it is not surprising to see increasing overall disease burden observed in our study (19).

Importantly, our study showed that the incidence of osseous metastasis between 2017 and 2018 is 28% in FLO and 29% in SLB, with increasing rates of bone metastasis across the treatment eras: FLO (23% vs. 24% vs. 28%; P = 0.0002) and SLB (15% vs. 25% vs. 29%; P < 0.0001). The presence and number of bone metastases have been associated with worse outcomes in patients with mRCC (20–23). Skeletal-related events (SREs) from osseous lesions can cause devastating complications such as bone fractures, spinal cord compressions, decreased quality of life, and significantly worsened outcomes. The National Comprehensive Cancer Network guidelines only recommend bone scans in patients with symptoms and/or an elevated alkaline phosphatase level, as prior studies did not support its routine use in RCC (7, 24, 25). Furthermore, osteolytic bone lesions in advanced RCC tend to evade the detection on a bone scan ^26^. Although CT scan of the chest, abdomen, and pelvis can potentially detect bone metastases, limited imaging field and heterogeneity in radiologic interpretations makes capturing such lesions more difficult. Higher expected percentage and increasing incidence of bone metastases in mRCC and their subpar detection by CT and bone scans, it is reasonable to consider routine whole-body skeletal survey in patients with mRCC, with fluorodeoxyglucose-PET or whole-body MRI-PET as other alternatives (26, 27). Timely detection can lead to early intervention, such as the targeted radiotherapy or the incorporation of bone targeting agents known to decrease SREs (28, 29).

Our study has several limitations because of its design and methodology. First, this study only focused on a clinical trial of patients with mRCC, and its applicability to patients in the real world may be limited. Second, the evolving RECIST definitions from 2000 and 2009 may have influenced the observed patterns. Third, the use of cross-sectional imaging routinely and the imaging quality have changed over the decades involved, which could contribute to the metastatic differences. The lack of consistent reporting on what radiographic modalities were used to assess patients before the study inclusion prevents us from elucidating specific trends in the frequency and modality of metastatic evaluation. Fourth, the settings (FLO vs. SLB) for the metastatic distribution were not clear for the selective studies involving the MIX setting. A “clean” analysis was performed wherein the MIX reports were excluded to ensure consistency. The “clean” analysis (data not shown) outcomes were not different from those reported here. Fifth, most samples were clustered in the VEGF-TKI Era, and data from several large clinical trials in the ICI-TKI Era were unavailable during the data analysis. There is also no clear-cut “year” for classification as the ICI-TKI era. But 2017 was selected as the cutoff as nivolumab, ipilimumab, pembrolizumab, and others were approved. Because of the overlaps between the VEGF-TKI and ICI-TKI Eras adoption in clinical practice, the difference before and after 2017 may not reflect the effects of therapeutic advancement. Sixth, most of the studies included were in patients with clear cell carcinoma. Some of the other subtypes and/or mixed histology were also included. The histologic and biologic behavior of nonclear cell carcinoma may be different from those of clear cell carcinoma, and studies to characterize metastatic site distribution of the former should be considered. Lastly, our study only examined metastasis to the lung, liver, bone, and LNs in mRCC. The lack of consistent metastasis reporting in other visceral sites such as the brain (ineligible in most studies) and adrenal glands (inconsistent reporting) was not possible because of the limited data availability.

## Conclusions

This study showed that the metastatic burden of disease in patients with mRCC has increased over the past three decades, with rising rates of lung, LN, and bone metastases. Given the unexpected high and rising rate of bone metastasis in this patient population, the use of clinically appropriate bone imaging should be considered as a diagnostic standard.
